# Physiological and Proteomic Analyses of Different Ecotypes of Reed (*Phragmites communis*) in Adaption to Natural Drought and Salinity

**DOI:** 10.3389/fpls.2021.720593

**Published:** 2021-09-13

**Authors:** Huan Li, Wen-Fang Lin, Zhi-Jun Shen, Hao Peng, Jia-Jie Zhou, Xue-Yi Zhu

**Affiliations:** ^1^Key Laboratory of the Ministry of Education for Coastal and Wetland Ecosystems, College of the Environment and Ecology, Xiamen University, Xiamen, China; ^2^College of Food and Bio-Engineering, Bengbu University, Bengbu, China; ^3^College of Life Science, Fujian Agriculture and Forestry University, Fuzhou, China; ^4^Department of Life Science and Engineering, Jining University, Jining, China

**Keywords:** reed, drought, salinity, anatomic, physiology, proteomic

## Abstract

Drought and salinity are the two major abiotic stresses constraining the crop yield worldwide. Both of them trigger cellular dehydration and cause osmotic stress which leads to cytosolic and vacuolar volume reduction. However, whether plants share a similar tolerance mechanism in response to these two stresses under natural conditions has seldom been comparatively reported. There are three different ecotypes of reed within a 5 km^2^ region in the Badanjilin desert of Northwest China. Taking the typical swamp reed (SR) as a control, we performed a comparative study on the adaption mechanisms of the two terrestrial ecotypes: dune reed (DR) and heavy salt meadow reed (HSMR) by physiological and proteomic approaches coupled with bioinformatic analysis. The results showed that HSMR and DR have evolved C_4_-like photosynthetic and anatomical characteristics, such as the increased bundle sheath cells (BSCs) and chloroplasts in BSCs, higher density of veins, and lower density and aperture of stomata. In addition, the thylakoid membrane fluidity also plays an important role in their higher drought and salinity tolerance capability. The proteomic results further demonstrated that HSMR and DR facilitated the regulation of proteins associated with photosynthesis and energy metabolism, lipid metabolism, transcription and translation, and stress responses to well-adapt to the drought and salinity conditions. Overall, our results demonstrated that HSMR and DR shaped a similar adaption strategy from the structural and physiological levels to the molecular scale to ensure functionality in a harsh environment.

## Introduction

Drought and salinity are the two major abiotic stresses affecting plant growth and constraining agriculture productivity because of their inhibitory effects on many physiological processes. Due to the sessile lifestyle, the plants often simultaneously suffer from various stresses in natural conditions. For example, in addition to water deficiency, plants growing in the desert regions are always accompanied by other stress factors as well, such as high temperature and high irradiance (Zhu et al., 2001, 2003c). A variety of evidence indicates that combined stress factors impact plant growth and development more severely than a single one (Pandey et al., 2017; Sengupta et al., 2021). Moreover, under natural conditions, stresses tend to develop gradually and progressively, and therefore, plant responses might be dramatically different from those abrupt stress treatments. However, the vast majority of experimental studies on plant responses to drought or salinity stress usually applied single artificial stress abruptly in a short period, although there are some data on the kinetics of drought and salinity treatments in the field plants (Wang et al., 2013; Li et al., 2017). The studies applied a single stress factor to help researchers to identify specific genes or/and proteins linked to the stress, but might not be able to illustrate the complex mechanism of plant responses to the multifactorial stress in natural conditions. So, it is necessary to comprehensively understand the response mechanisms formed in the adaption of plants to variable, multifaceted, and usually stressful natural conditions.

*Phragmites communis* is a hydrophytic species thriving across the world, whose typical habitats are fresh and brackish swamps, riverbanks, and lakesides. However, the reed can adapt to adverse terrestrial habitats, and various ecotypes exhibiting genetic differences have evolved resistance to drought and salinity (Matoh et al., 1988; Zhu et al., 2001, 2003a,b, 2012; Eller et al., 2017). Due to high intraspecific diversity and phenotypic plasticity, reed has an extensive ecological amplitude and a great capacity to acclimate to adverse environmental conditions, which therefore offers valuable insights into plant responses to natural stresses (Eller et al., 2017).

In northwest China, various ecotypes of *P. communis* with special adaptions to distinct habitats in the oasis-desert transitional zone have been investigated (Chen and Zhang, 1991; Zhu et al., 2003a,c). In addition to typical swamp reed (SR), the desert regions are home to two terrestrial reed ecotypes: dune reed (DR) and heavy salt meadow reed (HSMR). Since they are all located within a 5 km^2^ region, these three ecotypes of reed with a natural soil water potential gradient from wet to dry and a varied habitat salinity share similar meteorological conditions. Stable variations of morphological and physiological characteristics in response to drought and salinity and genetic diversity analysis on these reed ecotypes confirmed that they diverged from a common ancestor, which offers valuable insights into plants within one species in response to changing habitats with different soil water levels and soil salinity (Wang et al., 1998; Lin et al., 2007; Eller et al., 2017; Li et al., 2017). Over 20 years, comparative researches on these different ecotypes of reed in morphology, ultrastructure, physiological, and molecular distinctions have been extensively investigated (Chen and Zhang, 1991; Wang et al., 1998; Zhu et al., 2001, 2003b,c, 2012; Chen et al., 2003; Lin et al., 2007). These results show that water availability and soil salinity are important factors related to the high intraspecific diversity and phenotypic plasticity of these reed ecotypes; however, the specific mechanisms of two terrestrial ecotypes in adaption to drought and salinity habitats remain unclear.

To adapt to natural drought and salinity, plants develop several adaptive strategies at different levels, ranging from physiological through metabolic to molecular. Among these, the evolution of C_4_ photosynthetic characteristics is an important element. Way (2012) categorized two key traits primarily associated with the evolution of C_4_ photosynthesis as carbon-related traits (chemical limitation) and stomatal-related traits (stomatal limitation) concerning the ancestral C_3_ state. He pointed out that stomatal development might also be affected at the transition from C_3_ to C_4_ though there was no data available on when stomatal density or size was altered (Way, 2012). Different ecotypes of reed with a transition tendency of the photosynthetic pathway from C_3_- to C_4_-like may provide further information as a unique intraspecies system for studying changes in stomatal and chemical limitations along with the evolution of C_4_ expression.

Given that abiotic stress tolerance is a quantitative trait, and proteins are the true executors of physiological reactions in the cell, investigation on protein profiling in leaves of different ecotypes can also deliver substantial insights into the landscape of response to the stresses. Therefore, in this study, comparative proteomic analyses between the two terrestrial ecotypes reed (DR and HSMR) and the typical reed ecotype (SR) were also conducted to find out their adaption mechanisms. We aim to provide the biochemical and stomatal characteristics of the C_4_-like pathway in the two terrestrial ecotypes in adaption to natural drought and salinity. Obtained results will be helpful in fully understanding the mechanisms of plants in adaption to natural habitats, which combine multiple stress factors together.

## Materials and Methods

### Sampling Sites and Materials

**Figure 1:**
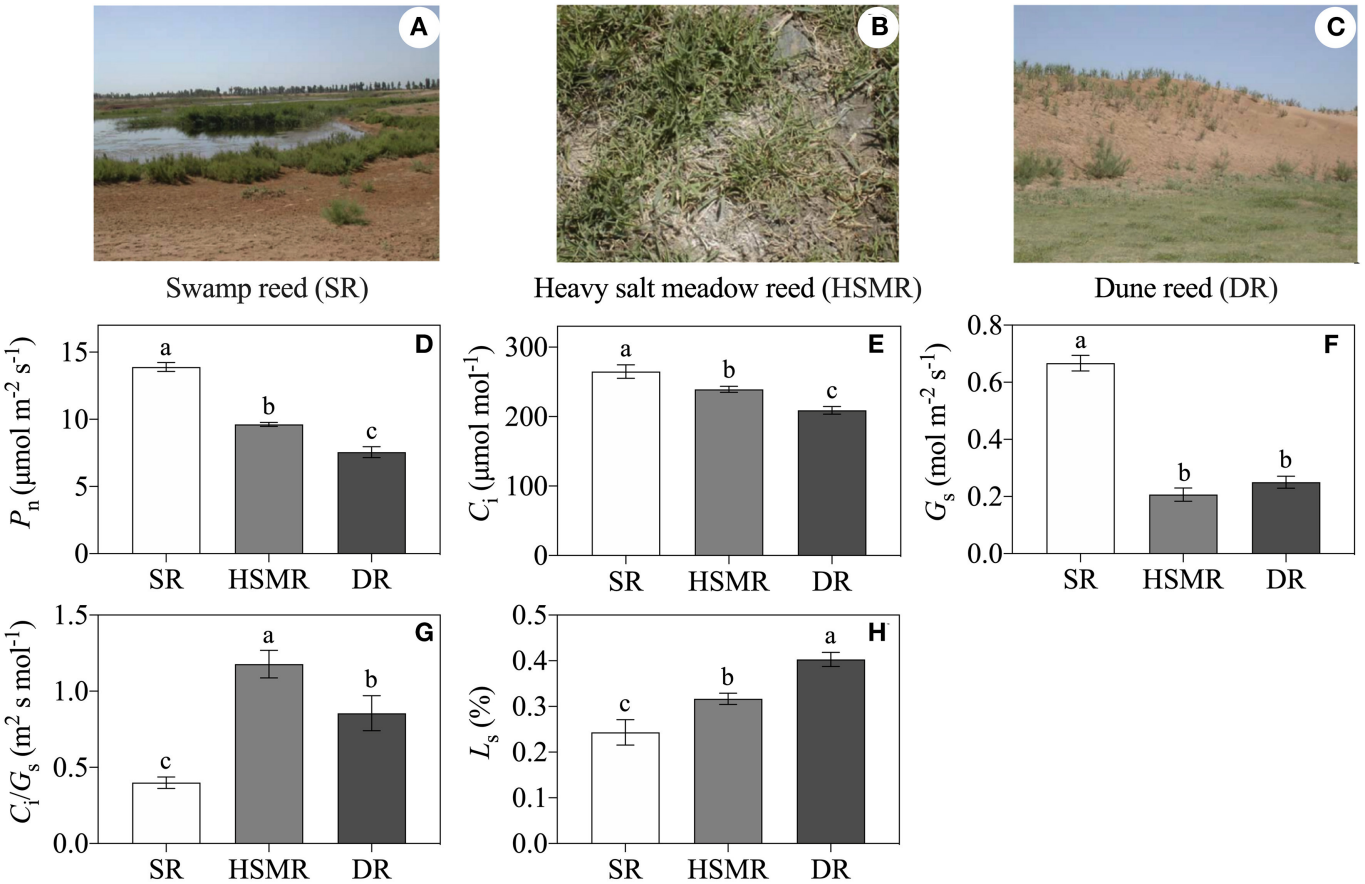
The habitats and physiological variations in three different ecotypes of reed. **(A)** Swamp reed (SR); **(B)** heavy salt meadow reed (HSMR); **(C)** dune reed (DR); **(D)** net photosynthetic rate (*P*_n_); **(E)** intercellular CO_2_ concentration (*C*_i_); **(F)** stomatal conductance (*G*_s_); **(G)** ratio of *C*_i_/*G*_s_; and **(H)** stomatal limitation (*L*_s_). Different lowercase letters at the data indicate significant differences among three different ecotypes of reed at p < 0.05 (one-way ANOVA). The photographs of three different ecotypes of reed were taken by Xue-Yi Zhu.

The study area is located near the Desert Research, Institute of Chinese Academy of Sciences, Linze, which belongs to the Hexi Corridor of arid and semiarid temperate desert regions of Northwest China. There is an oasis-desert transitional zone where different ecotypes of reed are distributed from swamp via heavy salt meadow to dune regions, building a natural soil water potential gradient from wet to dry and differential salinity habitats (Zhu et al., 2003c). Three reed ecotypes of *P. communis* referred to as SR, growing in the swamp with water depths varying from 1 to 2 m (39°22′8″ N, 100°7′49″ E, Figure 1A), HSMR, growing on the low-lying salt flats (39°22′48″ N, 100°7′18″ E, Figure 1B), and DR, growing on the 5- to 10-m-high sand dune (39°21′12″ N, 100°8′30″ E, Figure 1C). Since all sampling sites are located within a region of about 5 km^2^, they all share similar meteorological conditions. The second fully expanded leaves from the apex of the three reed ecotypes were individually sampled in early August. The leaves were frozen in liquid nitrogen immediately after harvesting and then stored at −80°C until extraction. Given the average depths of the fibrous root zone of the three ecotypes were 40, 60, and 15 cm, respectively, and soil samples were collected from the root zones of each ecotype accordingly. Salt content and moisture content in the root-zone soil were determined as our previous study (Zhu et al., 2003a). Meanwhile, leaves of C_3_ wheat and C_4_ maize growing in the local farming fields were also collected for leaf interveinal distance analysis.

### Gas-Exchange Measurements and Leaf Stoma Trait Comparisons

The second fully expanded leaves of the three ecotypes of reed were used for gas-exchange analysis. Net photosynthetic rate (*P*_*n*_), stomatal conductance (*G*_*s*_), and intercellular CO_2_ concentration (*C*_*i*_) were determined by an LI-COR 6400 portable photosynthesis system (LI-6400; LI-COR Inc., Lincoln, NE, USA) using the built-in light source set at 1,500 μmol m^−2^ s^−1^. The measurements were conducted under leaf temperature at 28 ± 2°C and 350 mmol mol^−1^ ambient CO_2_ concentration. Stomatal limitation (*L*_S_) was calculated based on the following equation (Shen et al., 2018):


$$\begin{array}{l} L_{s}=1-\left(C_{i}/C_{a}\right) \end{array}$$

Where *C*_a_ is the ambient CO_2_ concentration, while the ratio of *C*_i_/*G*_s_ is represented as the non-stomatal limitation.

Stoma distribution frequencies on the middle segments of the second leaves from the top of the three different ecotypes were used for analysis with the Phenom Prox Desktop SEM Generation 5 (Phenom-World, Netherland). The width and length of the stomatal aperture were measured by the Phenom-World Image Viewer software. The stomatal aperture of the middle segments of the second leaves collected from the top of three different ecotypes were calculated by the width:length based on the method of Takahashi et al. (2018). Presented data are the mean ± SE of measurements from the adaxial side of leaves of three individual plants.

### Measurements of Interveinal Distances in the Three Ecotypes of Reed, and C_3_ Wheat, and C_4_ Maize

Leaf interveinal distance was measured as described by Crookston and Moss (1974). Briefly, leaf middle segments of the second leaves from the apex were placed in 95% ethanol until the chlorophyll was extracted. The segments were then placed in a 10% aqueous sodium hydroxide (NaOH) solution and left overnight until they were cleared. The cleared segments were rinsed with distilled water and stained with I_2_-KI solution. Stained tissue was examined with a light microscope (Olympus, Japan). Determinations of vein frequency were made by measuring the actual distance between vein centers. Besides, wheat Dingxi 24 (*Triticum aestivum*) and maize variety Jiudan 3 (*Zea mays*) planting in local farm fields were collected for comparison to the typical C_3_ and C_4_ plants. Values represent averages of 6–10 separate determinations from three different plants of each ecotype.

### Determination of Chloroplast Distributions With Laser Confocal Microscope

For evaluating the number and size of the chloroplasts in bundle sheath cells (BSCs), fresh leaf segments (2 × 5 mm) were fixed in 2% glutaraldehyde buffered with 0.05 mol/l 1,4-piperazine-bis-ethanesulfonic acid (pH 7.2) overnight. After a series of ethanol dehydrated procedures, the specimens were treated with dimethylbenzene and then embedded in paraffin. Chloroplasts in the BSCs were observed with a laser scanning confocal microscope (Leica TCS-SP2-SE, Wetzlar, Germany). Chlorophyll autofluorescence was illuminated with 488 and 561 nm light separately. The numbers and sizes of the leaf BSCs were measured on confocal photomicrographs by the Motic Image advanced 3.0 software of the digital microscope (DBM-5, Global MoticGroup, Xiamen, China).

### Extraction and Determination of Fatty Acid

Fatty acid compositions in the isolated thylakoid membranes were assessed as described by Brown and Dupont (1989) with minor modification. Membrane lipids were extracted from the thylakoid membrane fractions with a mixture of chloroform and isopropanol. Isopropanol (1.06 ml) and chloroform (0.3 ml) were mixed with 0.4 ml of membrane fraction (300 μg from each sample) to form a monophasic solution followed by centrifugation at 1,000 × g for 3 min to precipitate proteins. Chloroform (1.83 ml) and 0.4 ml of 0.1 mol/l potassium chloride (KCl) were added to the supernatant to produce a biphasic solution. After thorough mixing, the phases were separated by centrifugation and the lower phase was washed three times with 1.0 ml aliquots of 0.1 mol/l KCl saturated with chloroform. The lower phase was dried under a stream of nitrogen (N_2_) and the lipids were dissolved in 0.5 ml of 0.4 mol/l potassium hydroxide (KOH) in methanol for methylation. The components of fatty acids were analyzed by gas chromatography (Shimadzu GC-9A, Japan). The assay conditions were as follows: FFAP quartz capillary column; hydrogen flame ionization detector (50 ml/min), air (50 ml/min); injector temperature: 260°C; column temperature: 200°C (3°C/min); carrier gas: N_2_; and spilt ratio: 100:1. The results were recorded with Shimadzu ZNP-R2A (Japan).

### Protein Extraction and Analysis of Two-Dimensional Gel Electrophoresis

Leaf proteins were extracted following our optimized phenol-ammonium acetate/methanol method (Lin et al., 2008). Briefly, leaf samples (0.5 g) were ground into tissue powder in liquid nitrogen and added 2.5 ml extracting solution containing 8 mol/l urea, 20 mmol/l DL-Dithiothreitol (DTT), and 2% NP-40. The mixture was transferred to centrifuge tubes followed by centrifugation at 15,000 × g for 3 min at 4°C. Then, the 1.2-fold volume of water-saturated phenol was added to the supernatant. The centrifuge tubes were turned upside down and gently shaken for 10 min, then separated by centrifugation at 10,000 × g for 20 min at 4°C. Proteins were precipitated from the phenol phase by the addition of five volumes of 0.1 mol/l ammonium acetate in methanol and incubated at −20°C overnight, then centrifuged at 28,000 × g for 3 min at 4°C. The precipitate was washed three times with the ammonium acetate in methanol and one time with methanol. The pellet was solubilized in lysis buffer consisting of 7 mol/l urea, 2 mol/l thiourea, 4% 3-[(3-cholamidopropyl) dimethylammonio]-1-propanesulfonate, 2% ampholine (pH 3.5–10:pH 5–8 = 1:4), and 65 mmol/l DTT in ultrasonic cleaner for 1 h at room temperature (RT), and then centrifuged at 10,000 × g for 2 min. The supernatant was collected and the protein concentration was measured using the Bradford method (Bradford, 1976).

Isoelectric focusing strips were made according to the method of Lin et al. (2008), which contained 3.5% ampholine (pH 3.5–10:pH 5–8 = 1:4). The negative electrode solution was 50 mmol/l NaOH and the positive electrode solution was 25 mol/l H_3_PO_4_. About 80 μg sample proteins were loaded for isoelectric focusing. Focusing was performed at RT of 200 V for 15 min, 300 V for 20 min, 400 V for 30 min, 500 V for 30 min, and 600 V for 16 h. The second sodium dodecyl sulfate-polyacrylamide gel electrophoresis (SDS-PAGE) was carried out with 12.5% acrylamide gels after the focused immobilized pH gradients (IPG) strips were equilibrated in 0.06 mol/l Tris-HCl (pH 6.8), 2% SDS, 100 mmol/l DTT, 10% glycerol, and 0.05% bromophenol blue for 30 min. Protein spots in analytical gels were visualized using silver nitrate staining protocol (Blum et al., 1987).

### Image and Mass Spectrometry Analysis

The 2-DE images were analyzed using PDQuest software (version 7.0, Bio-Rad, Hercules, CA, USA) for spot detection and protein quantification was performed according to Khan et al. (2018). Three gels of each sample (technical replicates) were considered as a whole to obtain a sample replicate set. Analysis of protein abundance was based on the changes in the relative spot volume. Only spots showing at least 1.5-fold variation in volume at *p* ≤ 0.05 level (Student's *t*-test) between different ecotypes of reed were considered as the differentially accumulated proteins (DAPs). The DAPs were manually excised from the gel, destained, and then dehydrated using acetonitrile. Tryptic digestion, peptide extraction, and mass spectrometry (MS) analysis were performed with MALDI-TOF MS Analyzer (Bruker, Germany) following the method of Khan et al. (2018).

Protein identification was carried out using the Mascot software (http://www.matrixscience.com) against taxonomy Viridiplantae (Green plant) in the NCBI non-redundant protein sequence database. The search criteria in the database were utilized following the method of Li et al. (2020). In addition, the criteria to obtain candidate proteins were set as follows: (i) the scores obtained from MOWSE must be over 60 (*p* < 0.05), (ii) at least three peptides matched, and (iii) the coverage of protein sequence has to reach a minimum of 10% (Li et al., 2020). Besides, the molecular weight, sequence coverage, and isoelectric point of detected proteins were also taken into consideration for comprehensively evaluating the candidate proteins (Li et al., 2020).

### Protein Classification, Hierarchical Cluster, and Protein-Protein Interaction Analysis

The functional classification of DAPs was searched in the databases of UniProt (http://www.uniprot.org) and NCBI (http://www.ncbi.nlm.nih.gov). The Venn diagram was drawn by the online software Draw Venn Diagram (http://bioinformatics.psb.ugent.be/webtools/Venn/). Hierarchical clustering analysis of DAPs was performed by using Cluster v3.0 and Treeview v1.1.3 software. The protein-protein interaction (PPI) network of DAPs was constructed by using String v11.0 (http://string-db.org). The PPI network was subsequently reconstructed by using Cytoscape v3.4.0 for data visualization and using plug-in MCODE to identify the core modules in the PPI network.

## Results

### Habitat Conditions, Phenotype, and Photosynthetic Parameters

As shown in Figures 1A–C and Table 1, although the three ecotypes of reed are located within 5 km^2^ and share similar meteorological conditions, they possess unique habitats and have significant differences in growth and development. Compared to the typical SR growing in the swamp with water-saturated soil and a 0.17% salt content in the root zone, the two terrestrial ecotypes (HSMR and DR) showed distinct differences in water and salt contents in the soil of the root zone, of which the HSMR growing on the low-lying salt flats had 43.53% water content and the highest salt content (0.86%) in the root zone, while the DR distributing on the 10 m high dune possessed the lowest water (18.37%) and salt contents (0.09%) in the root zone (Table 1). Morphologically, SR had a more than 3 m high shoot with the widest leaves followed by DR, and HSMR had <20 cm shoot with very narrow and small leaves (Table 1). The net photosynthetic rate (*P*_n_) measured around 10~11 a.m. was the highest in SR, followed by HSMR, and the lowest in the DR, which were closely related to their respective stomatal conductance (*G*_s_) and intercellular CO_2_ concentration (*C*_i_) (Figures 1D–F). The ratio of *C*_i_/*G*_s_ and the *L*_s_ in the two terrestrial ecotypes were observed higher than those in the SR (Figures 1G,H).

**Table 1:** Water and salt conditions and basic parameters of three ecotypes of reed.

**Basic parameters**	**SR**	**HSMR**	**DR**
Air temperature in canopy (°C)	31.2	33.1	32.6
Canopy relative humidity (%)	59.86	23.07	16.72
Soil water content (%)	Water saturated	43.53	18.37
Leaf water content (%)^*^	90.84	75.77	62.34
Soluble salt content in soil (%)	0.17	0.86	0.09
Shoot height (m)	3.23	0.18	1.36
Leaf width (cm)	5.21	1.17	2.13
Leaf length (cm)	45.38	3.57	25.15

*(A) SR, swamp reed; (B) HSMR, heavy salt meadow reed; (C) DR, dune reed*.

* *Data cited from Zhu et al. (2003a)*.

### Microstructure Analysis

**Figure 2:**
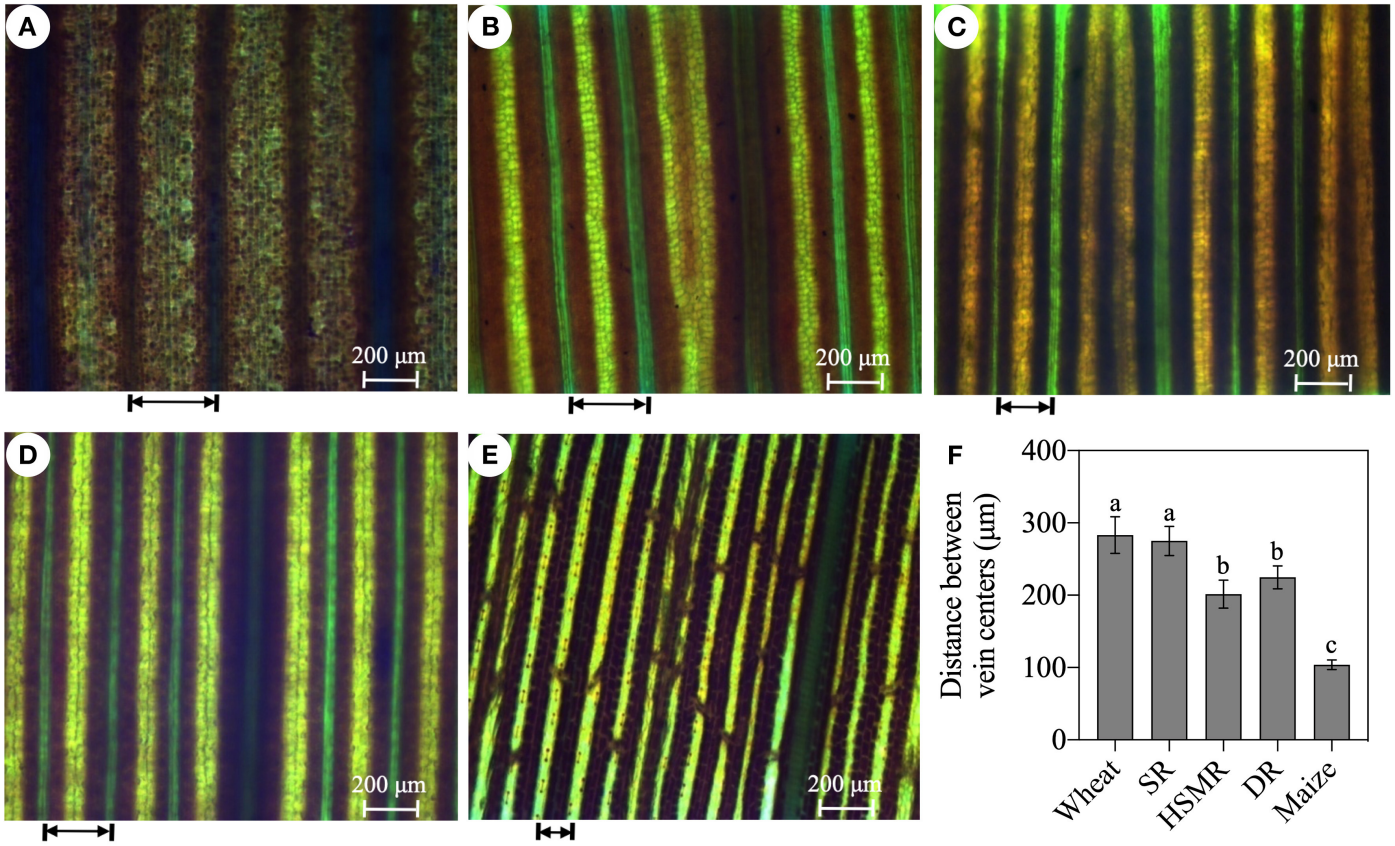
Leaf vein frequencies of the three reed ecotypes and the typical C_3_ wheat, the C_4_ maize. **(A)** Typical C_3_ wheat; **(B)** SR, swamp reed; **(C)** HSMR, heavy salt meadow reed; **(D)** DR, dune reed; and **(E)** typical C_4_ maize; and **(F)** the average distance between vein centers of five measured plants. The arrow represents the distance between the veins. Different lowercase letters at the data indicate significant differences among three different ecotypes of reed at *p* < 0.05 (one-way ANOVA).

**Figure 3:**
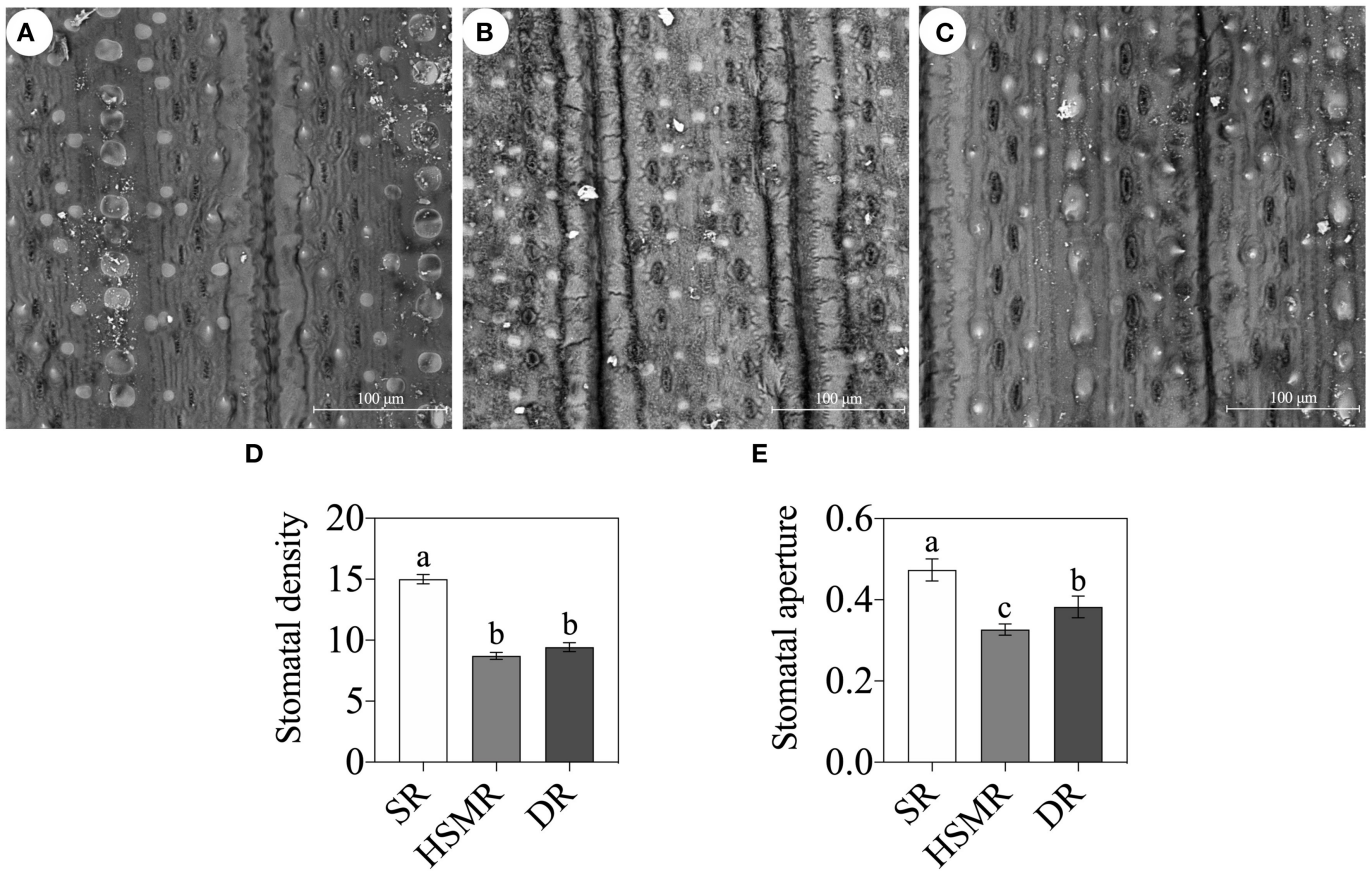
Scanning electron micrographs (800×) of the leaf surface of the three reed ecotypes (Bar = 100 μm). **(A)** SR, swamp reed; **(B)** HSMR, heavy salt meadow reed; **(C)** DR, dune reed; **(D)** stomatal density; and **(E)** stomatal aperture on leave surfaces of the three reed ecotypes. Different lowercase letters at the data indicate significant differences among three different ecotypes of reed at *p* < 0.05 (one-way ANOVA).

With the help of a light microscope, we found that the interveinal distances of the vascular bundles in the three ecotypes of reed were between the typical C_3_ wheat and C_4_ maize (Figure 2). In comparison with the swamp ecotype (SR), the two terrestrial ecotypes (DR and HSMR) showed markedly shorter interveinal distances (Figure 2). Furthermore, by using a laser confocal microscope, the structures of BSCs were clearly observed. The two terrestrial ecotypes (HSMR and DR) possessed smaller but more BSCs in comparison with SR. However, the two terrestrial ecotypes showed more and bigger chloroplasts along the centrifugal cell walls in their BSCs (Supplementary Figure 1). Given that the two terrestrial ecotypes possessed markedly shorter interveinal distances, relative numbers of chloroplasts in their BSCs were much more than that in SR in per unit leaf area. In addition, scanning electron micrographs of the leaf adaxial surfaces revealed that there were notable stomatal frequencies and distribution patterns among the three ecotypes of reed, of which HSMR and DR had lower stomatal densities and reduced stomatal apertures as compared with SR (Figure 3).

### Fatty Acid Compositions of Thylakoid Membrane

**Table 3** showed that palmitic acid was the major saturated fatty acid in the membrane lipid of thylakoid isolated in three ecotypes of reed, while linolenic acids were the predominant unsaturated fatty acids. For HSMR and DR, two saturated fatty acids, palmitic acid and stearic acid were significantly decreased by 47.72 and 20.45% and 5.13 and 22.76%, respectively, while linolenic acids (the major unsaturated fatty acids) were dramatically increased in the two terrestrial ecotypes by 63.54% in HSMR and 21.95% in DR as compared with SR. The indexes of unsaturated fatty acids (IUFA) were 51.89% higher in HSMR and 14.99% higher in DR.

### Protein Identification, Classification, and Hierarchical Clustering Analysis

**Figure 4:**
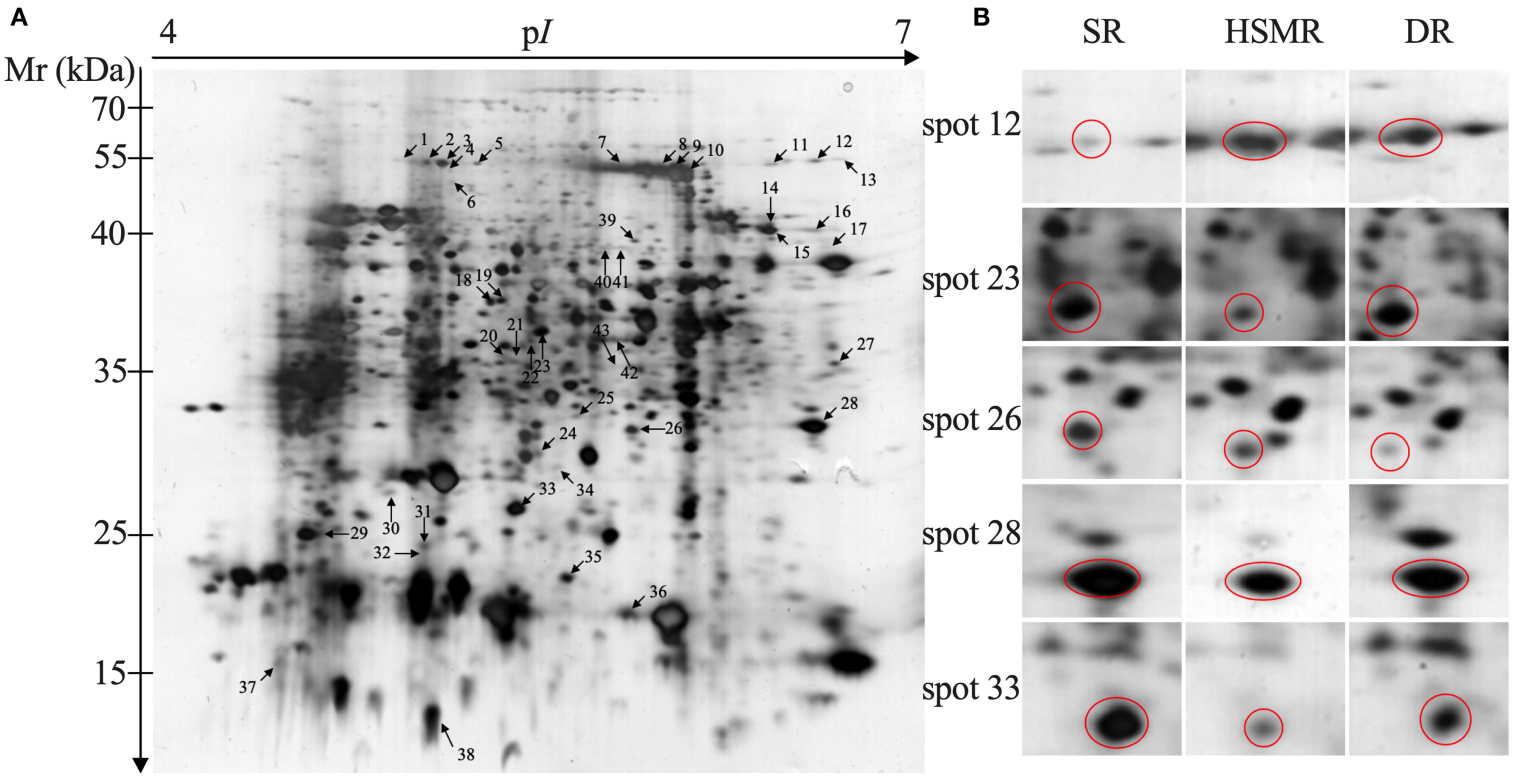
Two-dimensional electrophoresis analysis of protein extracted from three different ecotypes of reed. The numbers assigned to the spots correspond to those listed in Table 1. **(A)** Representative gel of total protein extracted from swamp reed (SR); **(B)** the enlarged windows of the representative spots with variation in protein abundance from three different ecotypes of reed.

**Figure 5:**
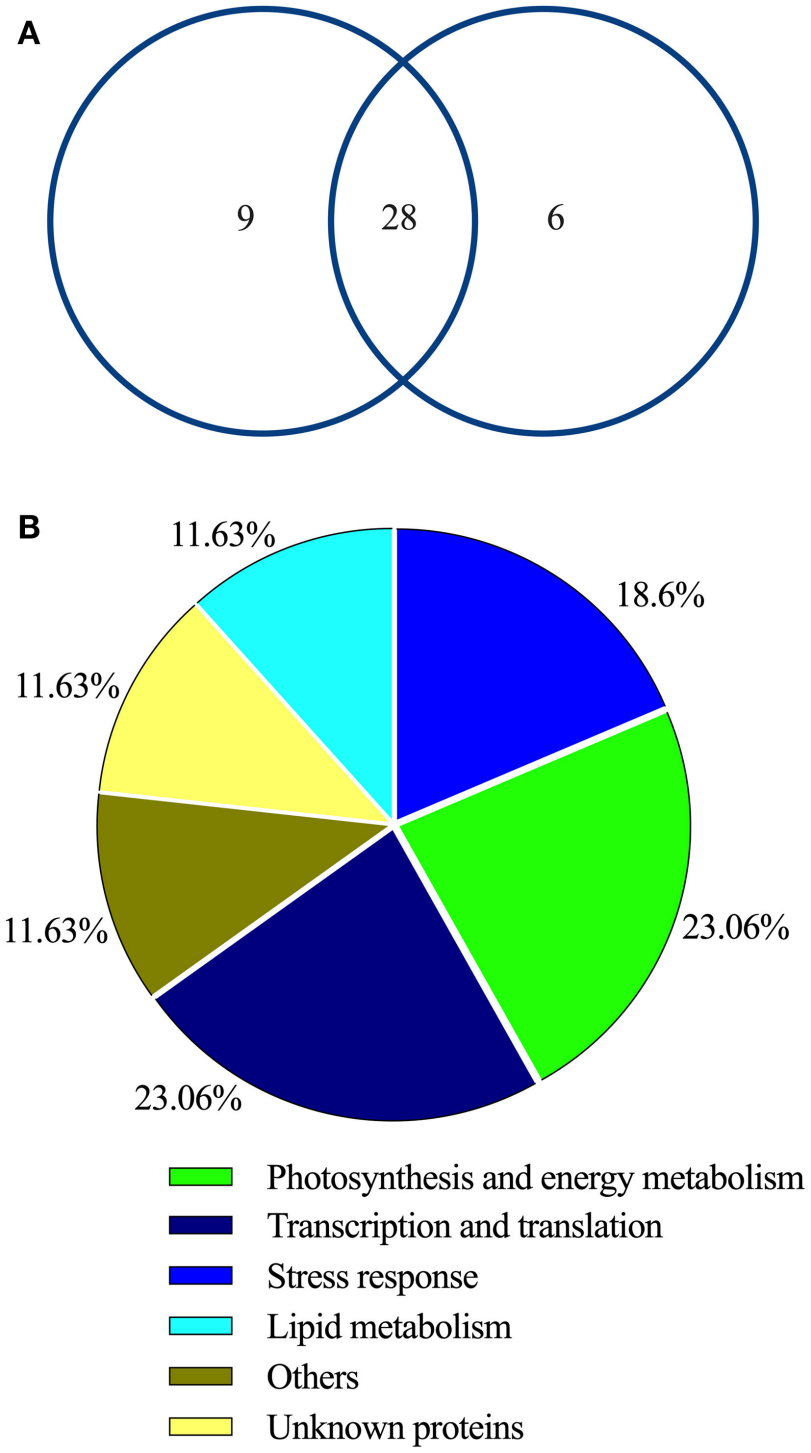
**(A)** The Venn diagram and **(B)** functional classification of 43 identified and quantified proteins from three different ecotypes of reed.

To explore the adaption mechanism of the two terrestrial ecotypes of reed under the long-term natural drought and salinity, 2-DE comparative proteomics was performed. A total of 43 spots were selected and successfully identified as DAPs (Figure 4 and Table 2). Among these DAPs, the abundances of 28 DAPs were changed in HSMR vs. SR and DR vs. SR. A total of nine and six DAPs were only observed in either HSMR vs. SR or DR vs. SR (Figure 5A).

**Table 2:** List of differentially accumulated proteins among the three reed ecotypes from various habitats.

**Spot^a^**	**Protein identify^b^**	**Accession (gb)^c^**	**Score^d^**	**Species**	**Ratio** ^**e**^	
					**HSMR vs. SR DR vs. SR**	
**Photosynthesis and energy metabolism**						
3	ATP synthase CF1 beta subunit	YP_009233529.1	133	*Centropodia glauca*	2.39	2.12
5	ATP synthase CF1 subunit beta	YP_003097583.1	111	*Dendrocalamus latiflorus*	3.19	4.49
9	RBCL, partial	AIF76083.1	88	*Halodule uninervis*	3.98	3.20
10	RBCL, partial	AOO78329.1	97	*Himalrandia lichiangensis*	39.63	24.38
14	RBCL, partial	AER28953.1	81	*Aponogeton undulatus*	2.00	1.90
23	Ribulose 1,5-bisphosphate carboxylase activase small isoform, partial	AIS19769.1	78	*Festuca pratensis*	0.33	0.88
24	Putative iron-sulfur cluster-binding protein	AAS07234.1	62	*Oryza sativa Japonica Group*	1.99	4.31
28	Ribulose bisphosphate carboxylase large chain precursor	AAK06652.1	84	*Mercurialis annua*	0.49	0.87
32	RBCL, partial	AOS89122.1	106	*Hamelia patens*	1.50	3.26
37	Digalactosyldiacylglycerol synthase 2	NP_191964.2	68	*Arabidopsis thaliana*	11.96	10.82
**Lipid metabolism**						
1	Cytochrome P450 94A1	KHN48177.1	94	*Glycine soja*	0.91	1.78
7	Acetyl-CoA carboxylase carboxyl transferase subunit beta, chloroplastic	PHT58582.1	73	*Capsicum baccatum*	2.69	1.20
15	Predicted: probable linoleate 9S-lipoxygenase 5	XP_009774053.1	73	*Nicotiana sylvestris*	1.54	1.10
26	NADH-cytochrome b5 reductase-like protein	XP_022730077.1	80	*Durio zibethinus*	0.49	0.11
42	Predicted: cytochrome P450 89A2-like	XP_011005629.1	65	*Populus euphratica*	1.29	2.70
**Transcription and translation**						
2	Retrotransposon-like protein	AQL09007.1	76	*Zea mays*	5.23	2.35
4	Zinc knuckle (CCHC-type) family protein	NP_567205.2	68	*Arabidopsis thaliana*	2.39	4.97
8	Maturase K, partial (chloroplast)	CCJ79218.1	81	*Arctium minus*	22.15	18.47
11	Predicted: DEAD-box ATP-dependent RNA helicase 27	XP_013629195.1	67	*Brassica oleracea*	8.49	8.90
16	Predicted: U11/U12 small nuclear ribonucleoprotein 25 kDa protein isoform X2	XP_017251432.1	75	*Daucus carota*	4.03	4.11
21	DEAD-box ATP-dependent RNA helicase 9	XP_008674301.1	71	*Zea mays*	1.55	2.07
30	Putative copia-like retrotransposon Hopscotch polyprotein	AAM18766.1	65	*Oryza sativa Japonica Group*	0.27	1.67
31	Predicted: U11/U12 small nuclear ribonucleoprotein 25 kDa protein isoform X2	XP_017251432.1	69	*Daucus carota*	1.20	1.52
36	Pentatricopeptide repeat-containing protein	PSS10437.1	61	*Actinidia chinensis*	2.47	2.31
43	Contains similarity to maize transposon MuDR	AAD49099.1	66	*Arabidopsis thaliana*	1.03	12.34
**Stress response**						
12	Catalase	NP_536731.1	106	*Oryza sativa*	3.32	3.25
19	Predicted: nucleoside diphosphate kinase	XP_015614147.1	68	*Oryza sativa Japonica Group*	0.17	0.45
29	Predicted: phosphatidylinositol/phosphatidylcholine transfer protein SFH11	XP_017223613.1	76	*Daucus carota*	4.26	4.68
33	Heat shock 70 kDa protein, mitochondrial-like	XP_020184128.1	76	*Aegilops tauschii*	0.22	0.35
34	Protein kinase 2B, chloroplastic	PKA55381.1	76	*Apostasia shenzhenica*	1.45	5.29
35	ABC transporter family protein	DQ103593.1	68	*Olimarabidopsis pumila*	0.50	1.12
40	Predicted: SKP1-interacting partner 15	XP_009104648.1	76	*Brassica rapa*	8.62	7.21
41	Predicted: alpha-dioxygenase 2-like	XP_019228527.1	68	*Nicotiana attenuata*	20.30	16.23
**Others**						
6	Predicted: phospho-2-dehydro-3-deoxyheptonate aldolase 1, chloroplastic isoform X1	XP_006363279.1	77	*Solanum tuberosum*	3.61	4.21
17	Predicted: indole-3-glycerol phosphate synthase, chloroplastic isoform X2	XP_018513205.1	64	*Brassica rapa*	14.65	21.21
25	Predicted: aspartate carbamoyltransferase 1	XP_016685011.1	90	*Gossypium hirsutum*	0.49	0.28
27	Structural maintenance of chromosomes protein 1, partial	AIU48096.1	72	*Aquilegia coerulea*	0.00	0.84
38	Predicted: deoxyuridine 5′-triphosphate nucleotidohydrolase	XP_006473483.1	82	*Citrus sinensis*	0.36	0.51
**Unknown proteins**						
13	Predicted: uncharacterized protein LOC102589741	XP_006343371.1	87	*Solanum tuberosum*	2.17	6.61
18	Uncharacterized protein LOC112006958	XP_023895045.1	82	*Quercus suber*	0.39	1.10
20	Hypothetical protein BC332_14714	PHU13509.1	84	*Capsicum chinense*	1.74	1.05
22	Predicted: uncharacterized protein LOC107279425	XP_015616476.1	66	*Oryza sativa Japonica Group*	1.94	4.34
39	Os03g0736600, partial	BAF13109.1	66	*Oryza sativa Japonica Group*	1.30	1.70

*SR, swamp reed; HSMR, heavy salt meadow reed; DR, dune reed*.

a *Spot numbers of differentially accumulated proteins*.

b *The names of the proteins identified by MALDI-TOF MS*.

c *Database accession numbers according to NCBInr*.

d *The Mascot searched score against the database NCBInr*.

e *Ratios of HSMR vs. SR and DR vs. SR*.

After searching the databases, these DAPs were categorized into six functional groups, including, photosynthesis and energy metabolism (23.26%), transcription and translation (23.26%), stress response proteins (18.6%), lipid metabolism (11.63%), others (11.63%), and unknown proteins (11.63%) (Figure 5B).

**Figure 6:**
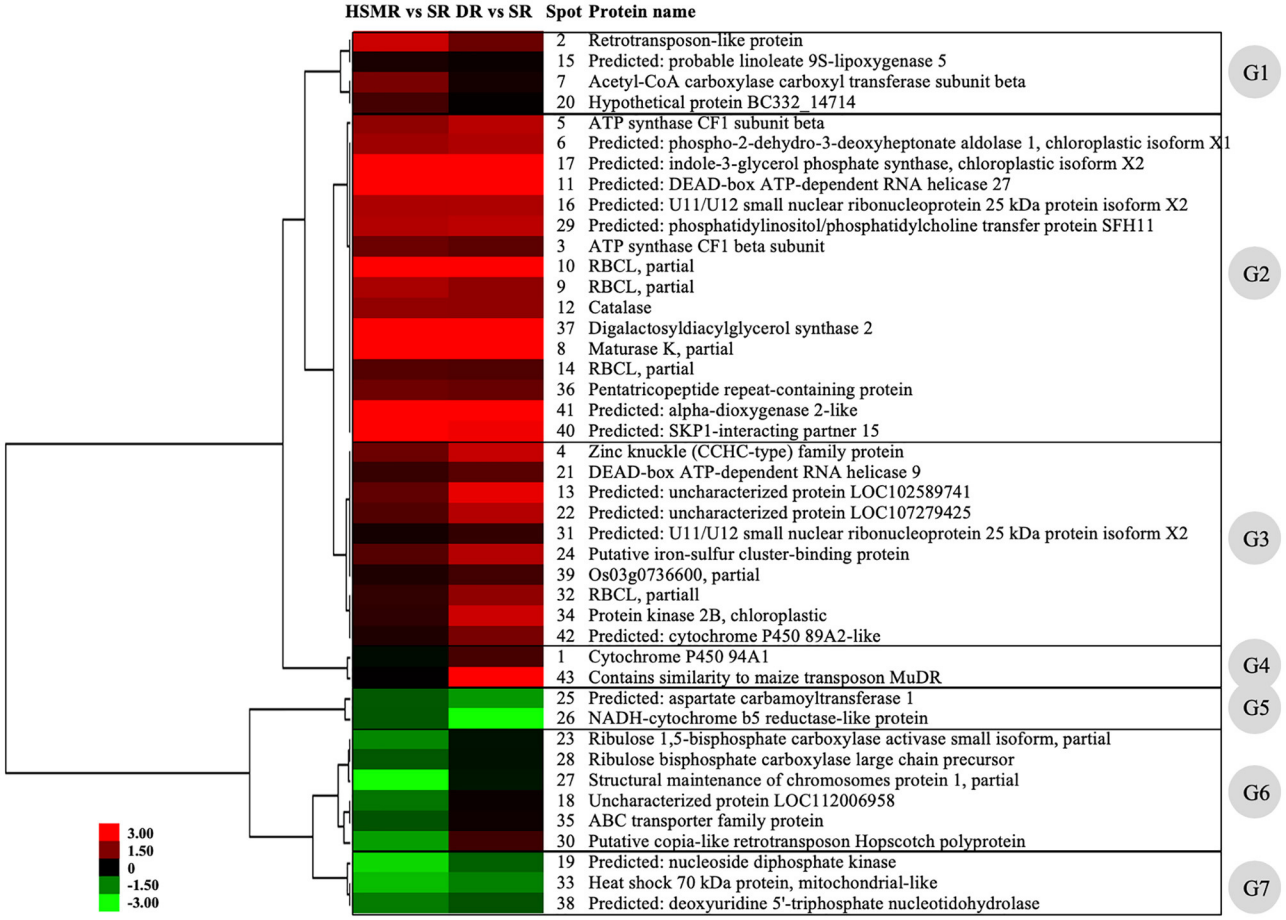
Hierarchical clustering analysis of the differentially accumulated proteins (DAPs) from three different ecotypes reed. The upregulated or downregulated proteins are presented in red or green. The intensity of colors stands for the abundance of protein as shown in the bar at the left of the figure. G1–G7 represent the seven clusters with different expression patterns among these DAPs.

Subsequently, hierarchical clustering analysis was performed to take a comprehensive overview of the expression patterns of these detected DAPs. Our clustering results showed that these 43 DAPs could be divided into seven groups (Figure 6). DAPs in group 1 (G1) were upregulated in HSMR vs. SR but no significant changes in DR vs. SR except spot 2, indicating that these DAPs were closely related with salt. DAPs in group 2 (G2) were significantly upregulated in both HSMR vs. SR and DR vs. SR, which implied that these proteins were involved in the adaption of reed not only to drought but also to salinity. Most DAPs in group 3 (G3) were also upregulated but they were much higher in DR vs. SR as compared to HSMR vs. SR. Two proteins in group 4 (G4) were only upregulated in DR vs. SR, indicating these two proteins were closely related to drought habitat. Both spots 25 and 26 in group 5 (G5) were downregulated and the ratios in DR vs. SR were lower than those in HSMR vs. SR. Similar to G5, three proteins in group 7 (G7) were also downregulated but their ratios in HSMR vs. SR were lower than those in DR vs. SR. DAPs in group 6 (G6) were only downregulated in HSMR vs. SR except spot 30, which was slightly upregulated in DR vs. SR.

### Protein-Protein Interaction Analysis

**Figure 7:**
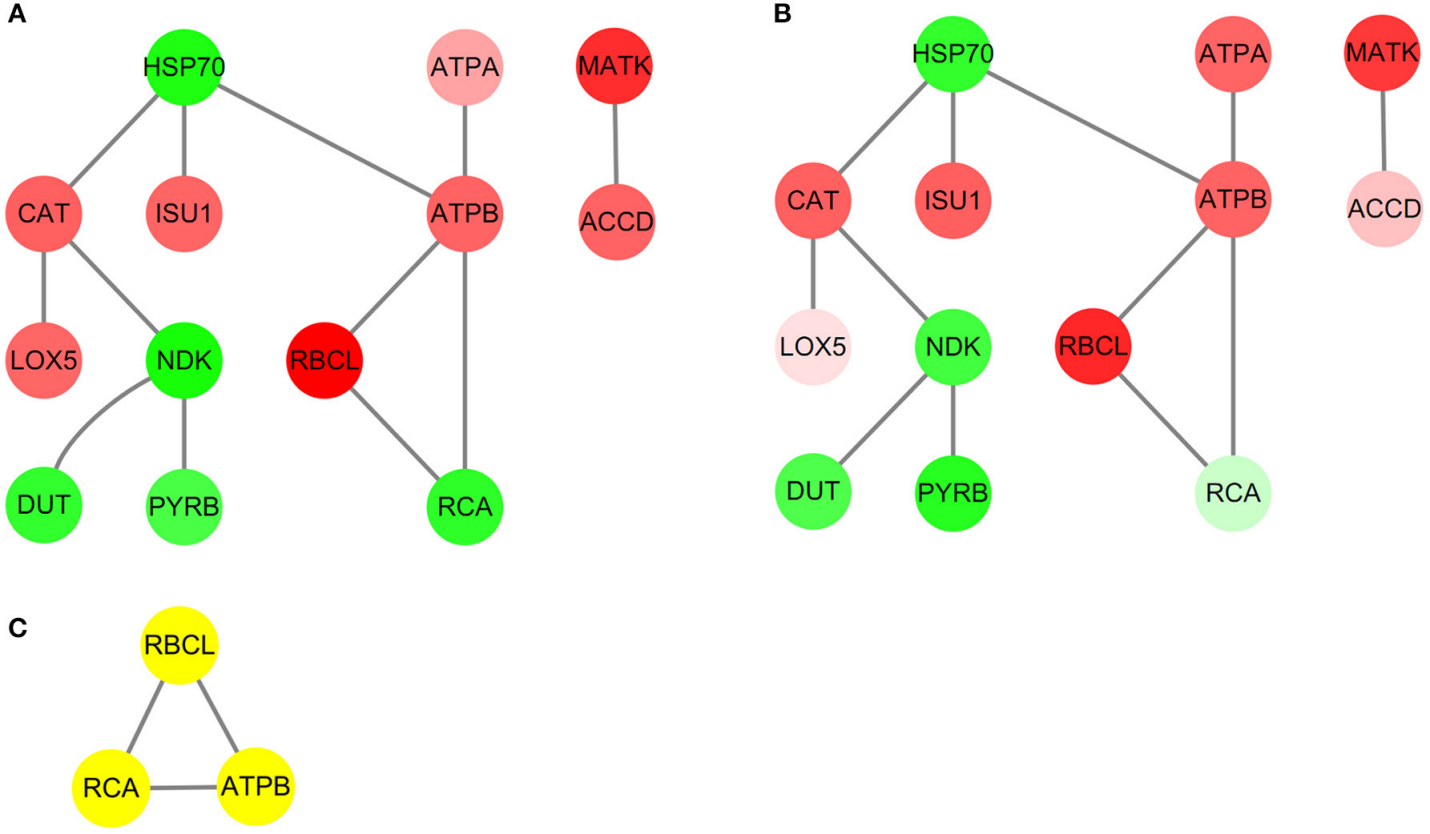
The protein-protein interaction (PPI) network analysis in the leaves of three different ecotypes reed. The upregulated or downregulated proteins are presented in red or green. **(A)** HSMR vs. SR; **(B)** DR vs. SR; and **(C)** result of clustering analysis in PPI network.

To further explore the functional modules among these three different ecotypes of reed, the PPI network was thus constructed (Figure 7). It showed that 13 proteins and 12 edges were successfully mapped in the String online database (Figures 7A,B). With the assistance of the MCODE plugin, the core subnetwork containing three proteins: ribulose-1,5-bisphosphate carboxylase/oxygenase (Rubisco) large subunit (RBCL), ribulose-1,5-bisphosphate carboxylase activase small isoform (RCA), and ATP synthase CF1 beta subunit (ATPB) was isolated from the PPI network (Figure 7C).

## Discussion

### Adaption Strategy in Photosynthesis and Energy Metabolism

As a plant species living in different habitats for a long time, the different groups will evolve some special morphological and physiological features to adapt to the variation in the habitats. Our fieldwork was carried out in a narrow region of 5 km^2^ in Northwest China, but the habitat conditions of the three different ecotypes of the reed were quite different (Figures 1A–C and Table 1). As a typical hydrophytic species, SR grows in a swamp area with perpetual water and has the highest water content in leaves (Table 1). In contrast, for the two terrestrial ecotypes, DR grows on the 10 m high dune with the lowest moisture content and HSMR grows on the salt flat with the highest salt content in the soil of the root zone (Table 1). This indicated severer drought stress in DR and heavier salt stress together with weaker drought stress in HSMR.

The arid and saline conditions usually bring about adverse effects on plant growth and development, and the reduction of photosynthesis (*P*_n_) was one of the important events of plants to drought and salt stresses (Shen et al., 2015). As expected, *P*_n_ was dramatically depressed in the two terrestrial ecotypes as compared to SR (Figure 1D). It was demonstrated that the stomatal limitation and/or non-stomatal limitation could affect *P*_n_ and CO_2_ assimilation of plants (Bellasio et al., 2018). In the present study, under natural drought and salinity environments, the stomatal densities, stomatal conductance (*G*_s_), and intercellular CO_2_ concentration (*C*_i_) in the terrestrial ecotypes were reduced, while their stomatal limitations (*L*_s_) were elevated (Figures 1, 3). Clearly, in adaptation to long-term natural drought and salinity, the two terrestrial ecotypes of reed regulated the rate of gas exchanges by adjusting their *G*_s_ through changes in stomatal density and aperture, which regulated evapotranspiration. These results implied that the stomatal limitation was one of the reasons to cause *P*_n_ decline both in DR and HSMR. Besides, it was suggested that the ratio of *C*_i_/*G*_s_ was an effective parameter to determine the non-stomatal limitation (Shen et al., 2018). In this study, higher *C*_i_/*G*_*s*_ ratios in DR and HSMR could reflect the involvement of non-stomatal factors in photosynthesis (Figure 1G).

Disadvantageous environmental factors, such as high temperature and radiation, low moisture, salinity, and low CO_2_, promoted C_4_ evolution in the plant photosynthesis (Osborne and Sack, 2012; Schlüter et al., 2017). The Kranz anatomy and high density of veins in leaves were the typical structures of the C_4_ plants (Schlüter and Weber, 2020). *P. communis* was a C_3_ hydrophytic species, however, investigations of leaf structures, δ^13^C, and C_4_ photosynthetic enzymes in different ecotypes of reed showed an obvious evolution tendency from C_3_ to C_4_ in adaptation to long-term natural drought and salinity habitats (Zheng et al., 1993; Zhu et al., 2012). Consistent with the previous studies, our confocal laser scanning micrograph results confirmed a Kranz anatomy-like structure with mesophyll cells and chloroplast-containing BSCs, especially in DR and HSMR, they both had more and bigger chloroplasts in their BSCs in comparison with the swamp ecotype, SR (Supplementary Figure 1). Moreover, comparative analysis of the leaf vein frequency and the distance between veins in the typical C_3_ wheat, the C_4_ maize, and the three ecotypes of reed exhibited gradual changes in anatomical features from C_3_ to C_4_ (Figure 2). These results implied that HSMR and DR could evolve more effective C_4_-like photosynthetic and anatomical characteristics to well-adapt to their natural adverse habitats.

Our proteomic results further confirmed the aforementioned results as well. Of which several DAPs associated with photosynthesis were identified (Table 2). Rubisco is an important enzyme in the Calvin cycle to fix CO_2_ into organic compounds (Andersson and Backlund, 2008). As a large subunit of Rubisco, RBCL was reported to evolve into a high-efficiency C_4_-like Rubisco that could enhance the photosynthesis of the C_3_ plant (Christin et al., 2008). In the present study, the abundances of RBCL were increased both in HSMR vs. SR and DR vs. SR (spots 9, 10, 14, and 32) (Table 2). This coincided with the conclusion obtained from subterranean clover plants grown under field conditions and subjected to progressive water stress, i.e., long-term water stress did not reduce the amount of Rubisco protein but decreased its activity (Medrano et al., 1997). This also implied the importance of Rubisco at the level of post-translational regulations.

Compared with the C_3_ photosynthetic pathway, the C_4_ pathway consumes more energy during the photosynthesis process (Katona et al., 1992; Zhu et al., 2003c). As the core catalytic site of the ATP production from the proton gradient across the membrane (Li et al., 2020), ATPB (spots 3 and 5) was successfully identified in this study, and both of them were upregulated (Table 2). This result also coincided with our previous work (Zhu et al., 2001). It was reported that the accumulation of ATPB could not only provide the extra energy required for biological processes of C_4_-like photosynthesis but also benefit ribulose biphosphate synthesis (Tezara et al., 1999). This might be one of the important adaptation mechanisms in the transition from C_3_- to C_4_-like pathway in these two terrestrial ecotypes.

The PPI network (Figure 7) and the core subnetwork analysis also verified our aforementioned conclusions (Figure 7C and Supplementary Table 1). Among these obtained DAPs (Figure 7C), RBCL and RCA were well-known to take roles in the Calvin cycle, while ATPB participates in energy production, which implied a close relationship between these two metabolic pathways. The functions of RBCL and ATPB were mentioned above and both of them were upregulated. RCA mainly functioned in activating the enzyme activity of Rubisco and was proved to have a negative relationship between RCA and Rubisco contents (Fukayama et al., 2018). As expected, the abundance of RCA decreased.

Based on the aforementioned and previous results, we suggested that suffering long-term natural drought and salinity, stomatal factors limiting photosynthesis existed in the two terrestrial ecotypes, however, their photosynthetic CO_2_ assimilations were partly restored by increasing metabolic functions, including interacting regulatory network among Calvin cycle, energy metabolism, and biochemical regulation of RCA, which benefited the growth and development of the two terrestrial ecotypes in their adaption to the harsh habitats.

### Adaption Strategy in Lipid Metabolism

Lipids are important components of plant membranes, especially in the photosynthetic membrane, and, importantly, affect membrane functions, so they play important roles in the mechanisms that allow plants to develop stress tolerance (Liang et al., 2005). In this study, we successfully identified several DAPs related to lipid metabolisms, such as cytochrome P450 94A1 (CYP94A1, spot 1), acetyl-CoA carboxylase carboxyl transferase subunit beta (CAC, spot 7), and linoleate 9S-lipoxygenase 5 (LOX5, spot 15) (Table 2). CYP94A1, which catalyzes the hydroxylation of various fatty acids, was reported to be involved in plant defense (Benveniste et al., 2005). CAC is a subunit of the acetyl-coenzyme A carboxylase complex that catalyzes the reaction of *de novo* fatty acid biosynthesis (Ke et al., 2000). LOX5, which catalyzes the hydroperoxidation of lipids, was reported to participate in plant growth and defense in *Arabidopsis* (Vellosillo et al., 2007). In this study, the protein abundances of CYP94A1, CAC, and LOX5 were upregulated in HSMR and/or DR in comparison with SR (Table 2). It could be concluded that the fatty acid biosynthesis and defense response in HSMR and DR might facilitate their adaption to salinity and drought environments.

In addition, it had been demonstrated that the tolerance of plants to drought and salinity is believed to rely on the inherent level of fatty acid unsaturation and/or the capability to maintain or adjust fatty acid unsaturation (Upchurch, 2008). Also, the degree of membrane unsaturation is thought to be one determining factor in adaptation to salt stress (Mansour et al., 2020). For example, in barley (*Hordeum vulgare* L.), the IUFA was higher in the salt-tolerant cultivar than in the salt-sensitive cultivar (Liang et al., 2005). Consistently, increased plasma membrane (PM) unsaturation in barley root accompanied with salt stress was considered to be of adaptive value for tolerance to salinity, as to elevate membrane fluidity (Yu et al., 2018; Mansour et al., 2020). In addition, an important function of enhanced unsaturated fatty acids, especially linolenic acid, in the PM lipids in response to saline conditions was suggested to serve as a sink to scavenge reactive oxygen species (ROS; Yu et al., 2020). In accordance, significant IUFA and linolenic acid contents were observed in HSMR in comparison with SR (Table 3), these data are in favor of the assumption that the change in the membrane fluidity is essential for salt adaptation of HSMR. In addition to salinity, drought is severe environmental stress that constraint plant growth and crop productivity (Shi et al., 2018). It is well-known that the dehydration of tissues was able to damage cell membranes (Ivanova et al., 2020). Thus, the maintenance of membrane integrity and stability is crucial for plant adaptation to drought. In the study of *Arabidopsis thaliana* (ecotype Columbia), the dehydration of leaves results in an elevated proportion of polyunsaturated fatty acids (Gigon et al., 2004). On the contrary, the amounts of linoleic acid and linolenic acid were reduced in the drought-sensitive species *Salvia officinalis* (Bettaieb et al., 2009) and *Carthamus tinctorius* L. (Hamrouni et al., 2001). In our study, palmitic acid and stearic acid (the two saturated fatty acids) were significantly decreased, whereas linolenic acid (the major unsaturated fatty acid) was increased in DR as compared with SR (Table 3), suggesting that DR could confer higher drought tolerance capacity through impact on the unsaturation degree of fatty acids, which most likely affects the fluidity of the thylakoid membranes.

**Table 3:** The fatty acid compositions (mol%) among the three reeds from various habitats.

**Parameter**	**SR**	**HSMR**	**DR**
Palmitic acid	19.76 ± 0.49^a^	10.33 ± 0.78^c^	15.72 ± 0.20^b^
Palmitoleic acid	2.76 ± 0.21^a^	2.11 ± 0.23^b^	1.81 ± 0.19^b^
Stearic acid	3.12 ± 0.15^a^	2.96 ± 0.08^a^	2.41 ± 0.05^b^
Oleic acid	11.08 ± 0.17^b^	11.65 ± 0.26^a^	11.6 ± 0.19^a^
Linoleic acid	9.55 ± 0.96^a^	9.45 ± 1.05^a^	7.04 ± 0.60^b^
Linolenic acid	49.7 ± 2.45^c^	81.28 ± 0.91^b^	60.61 ± 0.91^b^
^*^IUFA	182.04	276.50	209.32

*Different lowercase letters at the data indicate significant differences among three different ecotypes of reed at p < 0.05 (one-way ANOVA)*.

*SR, swamp reed; HSMR, heavy salt meadow reed; DR, dune reed*.

* *IUFA (index of unsaturated fatty acid) = 1 × %monenes + 2 × %dienes + 3 × %trienes*.

### Adaption Strategy in Transcription and Translation

The regulation of gene expression and protein biosynthesis is the most important defense response of plants against various stresses. Our proteomic data showed that eight DAPs were grouped into transcription and translation (Figure 5B), and most of them were more or less upregulated either in HSMR or DR as compared with SR (Table 2). Such as zinc knuckle (CCHC-type) family protein (spot 4), Maturase K (matK, spot 8), DEAD-box ATP-dependent RNA helicase (RH, spots 11 and 21), and pentatricopeptide repeat-containing protein (PNM, spot 36), all their protein abundances were increased in HSMR and DR (Table 2). CCHC-type family protein was the serine/arginine-rich protein that binding with RNA and probably participate in pre-mRNA splicing during RNA transcription (Aceituno-Valenzuela et al., 2020). matK was reported to control the gene expression in chloroplast by splicing (Barthet et al., 2020). RH had been proved to be involved in pre-mRNA splicing and was responsible for the transportation of mRNA from the nucleus to the cytoplasm (Kammel et al., 2013). Hammani et al. (2011) found that PNM interacted with nuclear proteins to regulate gene expression in the nucleus. Accumulation of these proteins indicated that both transcriptional and translational regulations played important roles for DR and HSMR in their adaption to natural drought and salinity.

### Changes in Stress-Related Proteins

As plants were exposed to drought and salt environments, the stress response proteins were induced (Zhang et al., 1992; Zhu et al., 2003c). Catalase (CAT, spot 12) mainly functioned in protecting cells from the damage caused by ROS (Alam and Ghosh, 2017). The higher abundances of CAT in both HSMR and DR suggested that CAT would be beneficial for HSMR and DR for protecting them from oxidative stress under drought and salinity. This was in accordance with our previous assessment in the enzyme activity of CAT (Zhang et al., 1992; Zhu et al., 2003c). SKP1-interacting partner 15 (SKIP15, spot 40) participated in E3 ubiquitin ligase-mediated ubiquitination and degradation of target protein (Gagne et al., 2002). The accumulation of SKIP15 to some extent both in HSMR and DR could lead to the degradation of some incorrect assemble proteins. Alpha-dioxygenase 2 (DOX2) was reported to highly express during the senescence process and microbial infection and hence could catalyze the oxygenation of fatty acids into oxylipins (Bannenberg et al., 2009). Of which oxylipins are involved in plant development and in response to biotic and abiotic environmental cues. Such as the functional convergence of oxylipin and abscisic acid pathways controls the stomatal closure of *Arabidopsis* in response to drought (Savchenko et al., 2014). Oxylipins are also acting to stimulate mitogen-activated protein kinase (MAPK)-dependent signaling pathways and induce the stress gene expression during salt stress (Hou et al., 2016). As a homologous protein of DOX2, the abundance of alpha-dioxygenase 2-like (spot 41) was increased in HSMR and DR (Table 2), suggesting that oxylipins mediation signaling cascades may contribute to the two terrestrial ecotypes tolerance to drought and salinity.

## Conclusion

**Figure 8:**
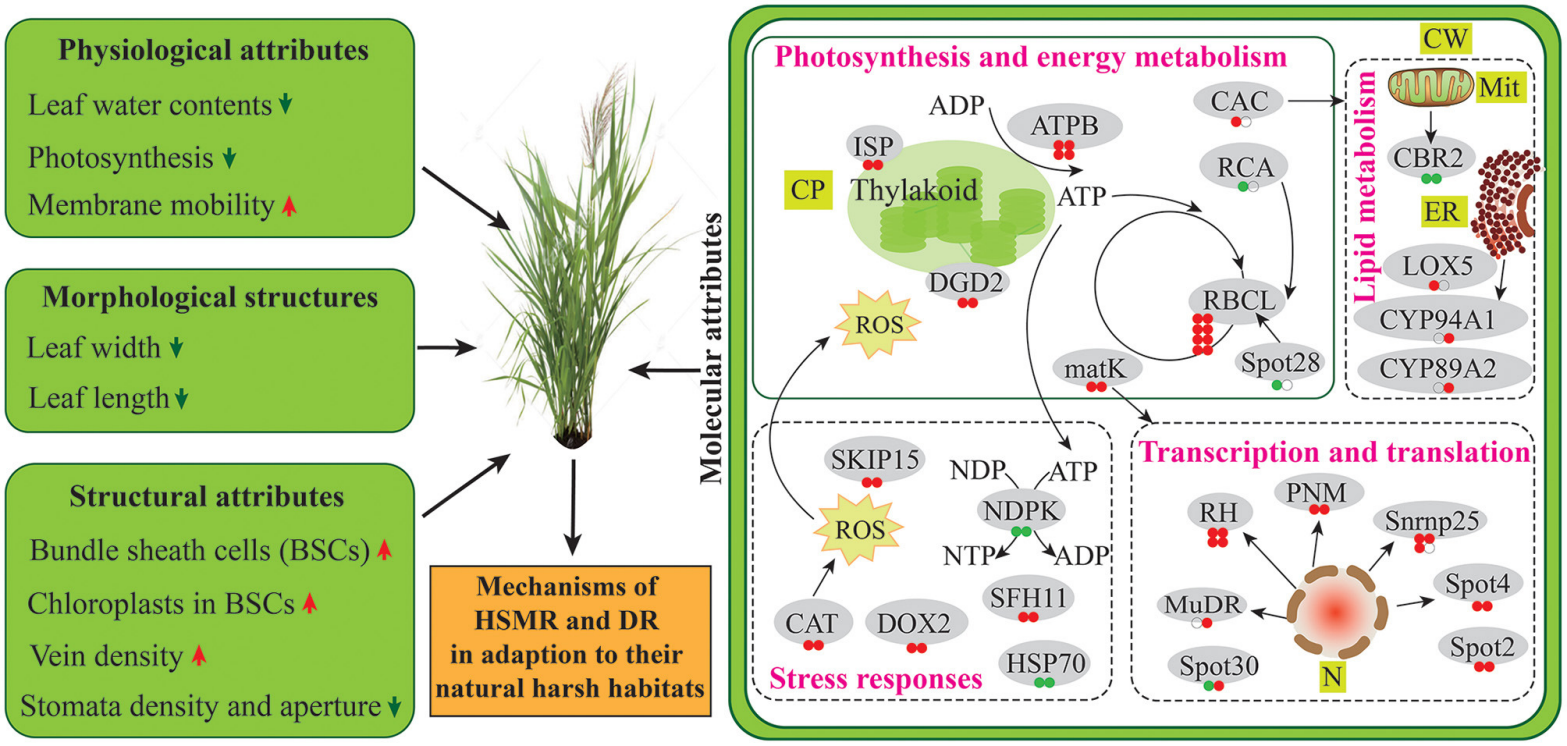
Schematic presentation of two terrestrial ecotypes in adaptation to their natural harsh habitat. Protein abundances are marked in circles. From left to right, the first and second circles represent HSMR vs. SR and DR vs. SR, respectively. Red color represents increased, green color represents decreased, and white color represents no change in protein abundance. ADP, adenosine diphosphate; ATP, adenosine triphosphate; ATPB, ATP synthase CF1 beta subunit; CAC, acetyl-CoA carboxylase carboxyl transferase subunit beta; CAT, catalase; CBR2, NADH-cytochrome b5 reductase-like protein; CP, chloroplast; CW, cell wall; CYP89A2, cytochrome P450 89A2; CYP94A1, cytochrome P450 94A1; DGD2, digalactosyldiacylglycerol synthase 2; ER, endoplasmic reticulum; HSP70, heat shock 70 kDa protein; ISP, putative iron-sulfur cluster-binding protein; LOX5, linoleate 9S-lipoxygenase 5; matK, maturase K; Mit, mitochondria; N, nucleus; NDP, nucleoside diphosphate; NDPK, nucleoside diphosphate kinase; NTP, nucleoside triphosphate; RBCL, ribulose-1,5-bisphosphate carboxylase/oxygenase large subunit; RCA, ribulose-1,5-bisphosphate carboxylase activase small isoform; RH, DEAD-box ATP-dependent RNA helicase; PNM, pentatricopeptide repeat-containing protein; SFH11, phosphatidylinositol/phosphatidylcholine transfer protein SFH11; Snrnp25, U11/U12 small nuclear ribonucleoprotein 25 kDa protein isoform X2; SKIP15, SKP1-interacting partner 15.

The possible tolerance mechanisms of reed growing in the natural drought and salinity habitats were comparatively analyzed in physiological and proteomic aspects in this study. Our results unraveled that the two terrestrial reed ecotypes: DR and HSMR, which are growing in extreme environments, both tended to shape similar adaptive strategies by impacting the formation of C_4_-like photosynthesis and anatomical features, lipid composition and mobility, as well as the protein abundances related to photosynthesis and energy metabolism, lipid metabolism, transcription and translation, and stress responses (Figure 8). Altogether, this species displayed a combination of multiple adaptive strategies from the structural and physiological levels to the molecular scale, to sustain the functionality of the two terrestrial ecotypes in their adaption to the hostile environments.

## Data Availability Statement

The datasets presented in this study can be found in online repositories. The names of the repository/repositories and accession number(s) can be found below: MassIVE [accession: MSV000087962].

## Author Contributions

X-YZ designed the experiments. W-FL, HL, HP, and J-JZ performed the experiments. HL and Z-JS analyzed the proteomic data. HL and X-YZ wrote the paper. W-FL, HP, and J-JZ gave the suggestions. X-YZ revised the paper. All authors have read and approved the manuscript.

## Funding

This work was supported by the NSFC (National Natural Science Foundation of China, No. 30470164).

## Conflict of Interest

The authors declare that the research was conducted in the absence of any commercial or financial relationships that could be construed as a potential conflict of interest.

## Publisher's Note

All claims expressed in this article are solely those of the authors and do not necessarily represent those of their affiliated organizations, or those of the publisher, the editors and the reviewers. Any product that may be evaluated in this article, or claim that may be made by its manufacturer, is not guaranteed or endorsed by the publisher.

## Supplementary Material

Supplementary Figure 1 Confocal laser scanning micrograph of chloroplasts in the bundle sheath cells from the paraffin section of three different ecotypes of reed. The chlorophyll fluorescence was illuminated with 488 nm (red images) and 561 nm (green images) light wavelength separately, the third column (yellow images) were their merged ones (bars = 30 μm). **(A–C)** Swamp reed (SR); **(D–F)** heavy salt meadow reed (HSMR); **(G–I)** dune reed (DR). BSC, bundle sheath cell; CP, chloroplast.

Supplementary Table 1 The detailed information of interaction network of the differentially accumulated proteins generated by STRING database.
