# Supplementary figures and images for: Physiological and Proteomic Analyses of Different Ecotypes of Reed (Phragmites communis) in Adaption to Natural Drought and Salinity
Source: Front Plant Sci. 2021 Sep 13;12:720593. doi: 10.3389/fpls.2021.720593 (PMC8473735; doi:10.3389/fpls.2021.720593)

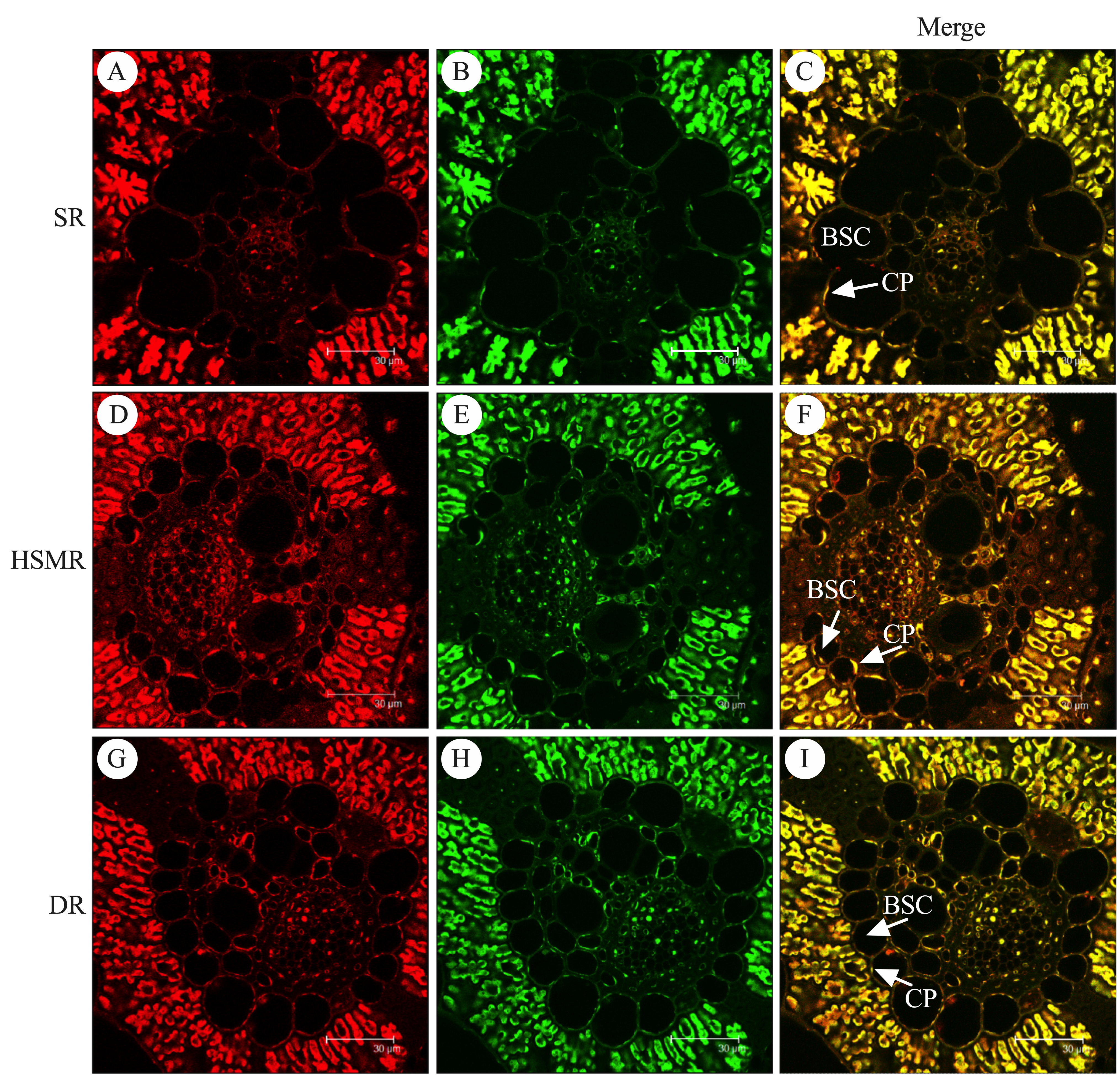

Supplement: Supplementary Figure 1 — Confocal laser scanning micrograph of chloroplasts in the bundle sheath cells from the paraffin section of three different ecotypes of reed. The chlorophyll fluorescence was illuminated with 488 nm (red images) and 561 nm (green images) light wavelength separately, the third column (yellow images) were their merged ones (bars = 30 μm). (A–C) Swamp reed (SR); (D–F) heavy salt meadow reed (HSMR); (G–I) dune reed (DR). BSC, bundle sheath cell; CP, chloroplast. [file Image_1.TIFF]
