# Supplementary material for: Physiological and Proteomic Analyses of Different Ecotypes of Reed (Phragmites communis) in Adaption to Natural Drought and Salinity
Source: Front Plant Sci. 2021 Sep 13;12:720593. doi: 10.3389/fpls.2021.720593 (PMC8473735; doi:10.3389/fpls.2021.720593)
Supplement: Supplementary Table 1 — The detailed information of interaction network of the differentially accumulated proteins generated by STRING database. [file Table_1.DOCX]

**Supplementary Table S1.** The detailed information of interaction network of the differentially accumulated proteins generated by STRING database.

| Node1 | Node2 | Neighborhood on  chromosome | Phylogenetic cooccurrence | Homology | Coexpression | Experimentally  determined interaction | Database annotated | Automated textmining | Combined  score |
| --- | --- | --- | --- | --- | --- | --- | --- | --- | --- |
| ATPB | ATPA | 0.123 | 0 | 0 | 0.778 | 0.701 | 0 | 0.454 | 0.963 |
| RBCL | ATPB | 0 | 0 | 0 | 0.189 | 0 | 0 | 0.937 | 0.947 |
| NDK | DUT | 0.066 | 0 | 0 | 0.134 | 0 | 0.9 | 0.407 | 0.945 |
| LOX5 | CAT | 0 | 0 | 0 | 0 | 0 | 0.9 | 0.075 | 0.903 |
| RCA | RBCL | 0 | 0 | 0 | 0 | 0 | 0 | 0.692 | 0.692 |
| ISU1 | HSP70 | 0.094 | 0 | 0 | 0.1 | 0.299 | 0 | 0.483 | 0.664 |
| PYRB | NDK | 0 | 0 | 0 | 0 | 0 | 0 | 0.632 | 0.632 |
| NDK | CAT | 0 | 0 | 0 | 0 | 0.341 | 0 | 0.419 | 0.601 |
| RCA | ATPB | 0 | 0 | 0 | 0.058 | 0.052 | 0 | 0.462 | 0.477 |
| CAT | HSP70 | 0 | 0 | 0 | 0.049 | 0 | 0 | 0.418 | 0.423 |
| ATPB | HSP70 | 0 | 0 | 0 | 0.147 | 0 | 0 | 0.333 | 0.406 |
